# Impact of lopinavir-ritonavir exposure in HIV-1 infected children and adolescents in Madrid, Spain during 2000-2014

**DOI:** 10.1371/journal.pone.0173168

**Published:** 2017-03-28

**Authors:** Patricia Rojas Sánchez, Luis Prieto, Santiago Jiménez De Ory, Elisa Fernández Cooke, Maria Luisa Navarro, José Tomas Ramos, África Holguín, Madrid Cohort of HIV-1 Infected Children and Adolescents Integrated in the Paediatric Branch of the Spanish National AIDS Network (CoRISPe)

**Affiliations:** 1 HIV-1 Molecular Epidemiology Laboratory, Microbiology and Parasitology Department, Hospital RamÓn y Cajal-IRYCIS and CIBER-ESP, Madrid, Spain; 2 Infectious Diseases Department, Hospital Universitario de Getafe, Madrid, Spain; 3 Molecular Inmuno-Biology Laboratory, Hospital Universitario Gregorio Marañón-IISGM and CIBER-BBN, Madrid, Spain; 4 Infectious Diseases Department, Hospital Universitario Doce de Octubre, Madrid, Spain; 5 Infectious Diseases Unit, Paediatric Department, Hospital Universitario Gregorio Marañón, Madrid, Spain; 6 Infectious Diseases Department, Hospital Clínico Universitario and Universidad Complutense, Madrid, Spain; Universita degli Studi di Roma Tor Vergata, ITALY

## Abstract

**Background:**

The most-used protease-inhibitor in children is Lopinavir-ritonavir (LPV/r), which provides durable suppression of viral load and increases CD4+T-counts. This study describes the virological outcome of the HIV-1-infected paediatric population exposed to LPV/r during 15 years in Spain.

**Methodology:**

Patients from the Madrid Cohort of HIV-1-infected-children and adolescents exposed to LPV/r as different line therapy during 2000–2014 were selected. The baseline epidemiological-clinical features, viral suppression, changes in CD4+T-CD8+T cell counts and drug susceptibility were recorded before and during LPV/r exposure. Drug resistance mutations (DRM) were identified in viruses from samples collected until 2011. We predicted drug susceptibility to 19 antiretrovirals among those carrying DRM using the Stanford′s HIVdb Algorithm.

**Results:**

A total of 199 (37.3%) of the 534 patients from the cohort were exposed to LPV/r during 2000–2014 in first (group 1), second (group 2) or more line-therapies (group 3). Patients were mainly Spaniards (81.9%), perinatally infected (96.5%) with subtype-B (65.3%) and HIV-diagnosed before year 2000 (67.8%). The mean age at first LPV/r exposure was 9.7 years. After protease-inhibitor exposure, viral suppression was higher in groups 1 and 2 than in group 3. Viral suppression occurred in 87.5%, 68.6% and 64.8% patients from groups 1, 2 and 3, respectively. Among the 64 patients with available resistance data during LPV/r treatment, 27(42.3%) carried DRM to protease-inhibitor, 28 (58.3%) to reverse-transcriptase-inhibitors and 21 (43.7%) to non-reverse-transcriptase-inhibitors. Darunavir/ritonavir, atazanavir-ritonavir and tipranavir/ritonavir presented the highest susceptibility and nelfinavir the lowest.

**Conclusions:**

A better lymphocyte recovering occurred when protease-inhibitor was taken as part of a first-line regimen and a higher number of patients reached viral suppression. The least compromised antiretrovirals for rescue antiretroviral regimens, according to DRM in the LPV/r-exposed-paediatric cohort, were mainly the new protease inhibitors.

## Introduction

By the end of 2014, three million children below the age of 15 years were HIV-infected [1]. The clinical outcomes in HIV-infected children have improved with effective combination antiretroviral therapy (cART) [2] that reduces the progression of HIV-1 disease and decreases AIDS-associated morbidity and mortality [3]. Early cART has superior clinical/immunological outcomes than deferred ART [4]. Treatment efficacy is compromised by an inappropriate adherence to treatment and by the selection of resistant virus [5]. Since approximately one-third of HIV-infected children experience virological failure within two years of initiating cART [6], drug resistance mutation (DRM) monitoring should be performed at diagnosis and after therapy failure to optimize first and second or more-line regimens. However, resistance studies in paediatric infections worldwide are still scarce [7].

According to international guidelines, first line cART for HIV-1-infected patients is a combination of two nucleoside reverse transcriptase inhibitors (NRTIs) and a third agent from a different class, either a non-NRTI (NNRTI) or a ritonavir-boosted-PI (PI/r) [8, 9]. The goals of PI/r are to reduce pill burden, side effects, drug interactions and medication cost and to preserve future treatment options [10]. LPV/r is the most-used PI in children today [11–15], and is the preferred antiretroviral (ARV) in first-line-cART for children less than 3 years old [16, 17] and for second-line cART for children when NNRTI-containing regimens were used in first-line cART [18]. LPV/r has been used as part of postexposure prophylaxis in infants [19]. LPV/r was approved in 2001 by the European Medicines Agency (EMA) [20] for children over 2 years and by U.S. Food and Drug Administration (FDA) in 2000 for infants older than 14 days [21]. An LPV/r-based regimen provides durable suppression of viral load (VL), increases CD4+ counts [22], and is more efficacious than a nevirapine (NVP)-based regimen in infants as first-line regimen [23], although it is less palatable. The aim of this study was to describe the virological outcome of HIV-1-infected paediatric population exposed to LPV/r as part of first, second, third or more cART regimens during 15 years in Spain.

## Materials and methods

### Study population

Patients from the Madrid Cohort of HIV-1-infected-children and adolescents exposed to LPV/r as different-line therapy during 2000–2014 were selected. Epidemiological information, clinical and virological data were recorded at baseline (before LPV/r exposure). We studied changes in viraemia, CD4+T-CD8+ lymphocyte T cell counts and DRM comparing baseline data and the last available determination during LPV/r experience until December 2014. Patients exposed to LPV/r as off-label therapy were also identified. The Madrid Cohort of HIV-1-infected-children and adolescents, established in 2003, includes the 36% of the infected paediatric population registered in Spain before the end of December 2014.

### HIV-1 *pol* sequencing

HIV-1 RNA extraction, amplification and sequencing were performed in *pol* coding region [protease (PR) and partial reverse transcriptase (RT), 1,121 bp] as previously reported [24]. New samples from 2 patients were provided by the Paediatric HIV BioBank integrated in the Spanish AIDS Research Network (RIS) RD12/0017/0035 and RD12/0017/0037 [25]. Samples were processed following current procedures and frozen immediately after their reception. All patients participating in the study gave their informed consent and protocols were approved by institutional ethical committees. The parents or legal guardians of all patients participating in the study gave their written informed consent to this BioBank, responsible for the storage of HIV-1 infected samples from the children under follow-up in Madrid used for this research. The project was approved by the Human Subjects Review Committee at Hospital Universitario Ramón y Cajal (Madrid, Spain).

### Accession numbers

HQ426735, HQ426744, HQ426750, HQ426756, HQ426760, HQ426761, HQ426773- HQ426776, HQ426778, HQ426783, HQ426790, HQ426793, HQ426796, HQ426800, HQ426830, HQ426833, HQ426839-HQ426842, HQ426844, HQ426849, HQ426856, HQ426883, HQ426884, HQ426894, HQ426901, JQ3519954, JQ351961, JQ351962, JQ351965, JQ351970, JQ351975, JQ351977, JQ351978, JQ351982, JQ351983, JQ351986, JQ351990, JQ351991, JQ351993, JQ351994, JQ351996, JQ352000-JQ352005, JQ352013, KP881486, KP881488, KP881497, KT318852- KT318873.

### Genotypic drug resistance identification

For the HIV drug resistance study, the patients were selected according to their *pol* sequence, genotypic resistance profile or sample availability by 2011. Most of these cases had been previously reported [24]. Two new *pol* sequences were obtained as previously described [26] from infected plasma samples provided by the HIV BioBank. DRM in accordance with the International AIDS Society-USA list 2014 (IAS-USA) [27], were expressed in percentages. Drug susceptibility to 19 antiretrovirals was predicted among those carrying DRM using the Stanford′s HIVdb Algorith(http://sierra2.stanford.edu/sierra/servlet/JSierra) in the last available PR sequence from patients carrying DRM, classifying drug susceptibility in four categories depending on mutation scores: susceptible, low-level, intermediate, and high-level resistance.

### Statistical analysis

For continuous variables, statistical difference based on the mean was calculated using t-tests. Variable distribution was described as median and interquartile range (IQR). Univariate and multivariate analyses were performed by Weka software (Waikato Environment for Knowledge Analysis, http://www.cs.waikato.ac.nz/ml/weka) to identify the risk factors for DRM development to PI.

## Results

### LPV/r experienced paediatric population

A total of 199 (37.3%) of the 534 patients from the Madrid Cohort of HIV-infected children and adolescents were exposed to LPV/r within the period 2000–2014. Their epidemiological and clinical features before first LPV/r experience are shown in Table 1 Most were Spaniards (81.9%), diagnosed before 2000 (67.8%), perinataly infected (96.5%) and carrying HIV-1 subtype-B. Last updated data showed that most (85.4%) were under follow-up in paediatric or adult units. The study population showed a mean age 3.2 years at first ART experience, with mono or dual therapy in 55. 3% of cases, a mean age at first LPV/r experience of 9.4 years and a mean time of LPV/ r exposure of 4.2 years.

**Table 1:** Baseline features of 199 LPV/r experienced children and adolescents from the Madrid cohort with (2000–2014).

Features	Number of LPV/r experienced patients (2000–2014)	As first ART regimen Group 1	As second ART regimens Group 2	As ≥ 3 ART regimens Group 3	With resistance data
**LPV/r use**^a^	199	33	37	126	64
**Female**	108	15	21	72	34
**HIV-1 transmission**					
Perinatal	192	29	37	123	63
Blood Transfusion	6	3	0	3	1
Unknown	1	1	0	0	0
**Origin**					
Spain / rest of Europe	163 / 2	19 / 0	27 / 1	117 / 1	57 / 0
Africa	20	13	3	3	5
North America	1	0	0	0	0
South America	13	1	6	5	2
**HIV-1 diagnosis period**					
1980–1989	16	0	0	16	8
1990–1999	119	0	16	102	39
2000–2013	64	33	21	8	17
**Mean age of diagnosis (months)**					
	24.2	31.9	35.1	19.4	26.7
	Range:0–144.9	Range:0–136.2	Range:0–144.5	Range:0–144.5	Range: 0–303
	IQR [2.9–36]	IQR [2.3–48.8]	IQR [2.2–59.1]	IQR [3.1–27.1]	IQR [2.9–30.5]
**Clinical follow up**					
Paediatric unit	90	26	21	41	31
Adults unit	80	0	8	72	24
Lost to follow-up/exitus	26 / 3	7 / 0	8 / 0	10 / 3	7 / 2
**HCV or HBV coinfection**	10 / 5	0 / 2	1 / 0	9 / 3	3 / 1
**Mean age at first ART regimen (months)**					
	38.8	35.2	42.5	40.1	41.3
	Range: 0–166.4	Range:0.9–137.7	Range:0.5–166.5	Range:0–300.5	Range: 0.9–172
	IQR [6.7–59.9]	IQR [3.3.-50.6]	IQR [6.2–79.5]	IQR [7.9–59.4]	IQR [6.6–65.2]
**First ART regimen**					
Monotherapy	67	0	0	67	24
Dual therapy	43	0	3	40	16
HAART	86	33	34	19	24
Unknown	3	0	0	0	0
**ART regimen including LPV/r**					
NRTI+PI	143	32	32	79	44
NNRTI+PI	3	1	0	2	20
NRTI+NNRTI+PI	48	0	5	43	0
NRTI+PI+CI	1	0	0	1	0
unknown	4	0	0	0	0
**Mean age at first LPV/r experience (months)**					
	113	35.2	101.8	136.8	104.5
	Range: 0.9–232	Range:0.9–138	Range:2.6–194	Range:10.8–232	Range: 0.9–207.5
	IQR [60.4–162]	IQR [3.3–50.1]	IQR [49.5–163.1]	IQR [107.6–178.8]	IQR [54.05–152.7]
**LPV/r use (2000–2007)** ^b^	164	18	28	118	59
**LPV/r use (2008–2014)** ^a^^,^^b^	35	15	9	8	5
**Mean time under LPV/r exposure (months)**					
	50.9	37.3	41.9	57.5	70.3
	Range: 0.1–106.5	Range: 0.9–106.5	Range: 0.1138.7	Range: 0.1–151	Range: 0.06–180
	IQR [19.2–80.5]	IQR [14.7-56-4]	IQR [13.8–64.7]	IQR [26.6–87.8]	IQR [28.5–97.5]
**With resistance data**	64	8	10	46	64
**LPV/r as off label**	31	14	4	13	7

^a^Unknown LPV/r use in 3 patients.

^b^Year for the first LPV/r usage in the study population (number of patients): 2000 (5), 2001 (32), 2002 (21), 2003 (23), 2004 (13), 2005 (20), 2006 (11), 2007 (39), 2008 (14), 2009 (5), 2010 (6), 2011 (2), 2012 (6), 2013 (2), 2014 (0). LPV/r, lopinavir-ritonavir; ART, antiretroviral treatment; HAART, highly active antiretroviral therapy; IQR, interquartile range; NNRTI, non nucleoside reverse transcriptase inhibitor; NRTI, nucleoside reverse transcriptase inhibitor; PI, protease inhibitor; CI, correceptor inhibitor. Since European medicine Agency approved LPV/r only in children older than 2 years, we considered the use of LPV/r as off—label antiretroviral when LPV/r was administered in children younger than 2 years old.

LPV/r was prescribed as cART in the 199 subjects, although in three (1.5%) of them the ART line was unknown. Among the remaining 196 pediatric patients, LPV/r was included in the first ART regimen in 16.6% patients (group 1), in the second line therapy in 18.6% (group 2), and in third or more line regimen in 63.3% (group 3). The mean time under LPV/r exposure was higher in patients from group 3 (4.8 years) compared with group 2 (3.5 years) or group 1 (3.1 years). The 64 children with available *pol* sequence or genotypic resistance profile showed a higher mean time under LPV/r experience (5.8 years). Since during the 2008–2014 period new alternative potent drugs for rescue ART regimens were approved for paediatric use by EMA, we studied LPV/r use in 2000–2007 *vs*. 2008–2014 periods. The rate of patients using LPV/r in the study cohort decreased over time (82.4% during 2000–2007 and 17.6% during 2008–2014) (Table 1). LPV/r was prescribed more frequently as part of first cART regimen during 2008–2014 than during 2000–2007 (42.8% *vs*. 11%). The use of LPV/r as off-label antiretroviral was frequent (15.6%) in our study population, as reported for the entire cohort [28].

### VL suppression after LPV/r exposure

Table 2 shows the rate of patients with viral suppression according to available VL data from baseline (before LPV/r exposure) in 180 (90.4%) subjects and during LPV/r experience in 194 (97.5%). A total of 3,117 VL measurements after first LPV/r exposure (range from 1 to 48 determinations per patient) were recovered from clinical reports (Table 3). As expected due to the absence of previous cART exposure, group 1 patients showed higher baseline VL values (5.8 log) than groups 2 and 3 (4.7 log each). Table 3 shows the rate of undetectable VL achieved under LPV/r exposure in the study population. Forty-six (25.5%) of the 180 patients with available baseline VL presented undetectable viraemia (VL< 500c/ml). They belonged to groups 2 (26.1%) and 3 (73.9%). However, nearly 70% of the 194 patients with available viremia data achieved undetectable VL (<500 HIV-1 RNA copies/ml) in the last available VL measurement (Table 3). Considering groups, ART including LPV/r achieved viral suppression (<500 HIV-1 RNA copies/ml) in 8.5%, 68.6% and 64.8% patients from groups 1, 2 and 3, respectively (Table 2). Group 1 also showed the highest rate of children with undetectable VL (<50c/ml) at last available measurement after LPV/r exposure compared to Group 2 (22/32, 68.5% *vs*. 17/35, 48.57%) (Table 3).

**Table 2:** Virologic and immunologic status of the complete study population with available data before and during LPV/r exposure.

Patients	Mean values	At baseline (Before LPV/r exposure)	During LPV/r exposure (last clinical report)	Δ	p-value
**Group 1**	**VL suppression**	0 patients^a^	87.5% patients^b^	-	-
	**CD4 cells**	1280.1; IQR [265–1906.2]^b^	1258.5; IQR [860.5–1692.7]^c^	-21.6	NS
	**CD4%**	23.2%; IQR [9–34]^b^	31.4%; IQR [24.4–38.3]^d^	+8.2%	0.03
	**CD8%**	33.7%; IQR [25.9–38.4]^b^	37.8%; IQR [37.8–46]^e^	+4.1%	0.03
**Group 2**	**VL suppression**	38.9% patients^f^	68.6% patients^g^	-	-
	**CD4 cells**	1220.1; IQR[604.5–1621.5]^f^	928.8; IQR [647.8–1194.1]^b^	-291.3	NS
	**CD4%**	28.9%; IQR [20.2–37.7]^f^	33%; IQR [25.5–39.4]^h^	+4.1%	NS
	**CD8%**	38.4%; IQR [30.1–45.9]^f^	40.8%; IQR [27–49]^i^	+2.4	NS
**Group 3**	**VL suppression**	28.8% patients^j^	64.8% patients^k^	-	-
	**CD4 cells**	733.8; IQR [426.5–952]^l^	790.7; IQR [551.7–1000.8]^m^	+56.9	NS
	**CD4%**	26.3%; IQR [18.1–35]^n^	30%; IQR [23.9–36.65]^o^	+3.7%	0.005
	**CD8%**	43.4%; IQR [34.9–51.1]^n^	45.5%; IQR [34.5–55.2]^p^	+2.1	NS
**Total**	**VL suppression**	2.8% patients^q^	19.6% patients^r^	-	-
	**CD4 cells**	898.5; IQR [438.7–1121.5]^s^	910.8; IQR [608.1–1186.6]^t^	+12.3	NS
	**CD4%**	26.4%; IQR [17–35]^u^	30.7%; IQR [23.99–37.59]^v^	+4.3%	0.0001
	**CD8%**	40.8%; IQR [32.5–48]^w^	43.7%; IQR [32–53.7]^x^	+2.9	0.05
**With resistance data**	**CD4 cells**	888.1; IQR [398–1118] ^Ω^	782.7; IQR [271–1033.5]^α^	-105.4	NS
	**CD4%**	26.5%; IQR [18–34.6]^μ^	25.4%; IQR [17.2–34.4] ^Ф^	-1.1%	NS
	**CD8%**	42.6%; IQR [30.75–53.1]^π^	47.2%; IQR [34.6–61]^α^	+4.6%	NS

Baseline, the last available data before LPV/r exposure. VL, virus load; IQR, interquartile range; Δ, difference between the available data before and during LPV/r experience. Rates calculated with available data in. 28^a^, 32^b^, 20^c^, 26^d^, 21^e^, 36^f^, 35^g^, 34^h^, 29i, 118^j^, 125^k^, 123^l^, 107^m^, 126^n^, 119^o^, 114^p^, 181^q^, 194^r^, 190^s^, 159^t^, 192^u^, 178^v^, 191^w^, 161^x^, 49^Ω^, 51^α^, 57^μ^, 54^Ф^, and 52^π^ HIV-1-infected children and adolescents from the study cohort. Significant differences when p<0.05. NS, not significant (p>0.05). VL suppression refers to patients with available VL data who reached non-detectable VL (<500 c/ml) post initiation of LPV/r in at least one determination during LPV/r exposure, even if they later rebounded.

**Table 3:** Undetectable viral load achieved under LPV/r exposure in the study population.

	Total^a^	Group 1	Group 2	Group 3	With resistance data
**Patients with available data**	194 (97.5%)	32 (97%)	37 (100%)	125 (99.2%)	64 (100%)
Mean time under LPV/r exposure (months)	50.9	37.3	41.9	57.5	70.3
Total number of VL measurements	3,117 (Range: 1–48)	438 (Range: 1–43)	572 (Range: 1–40)	2,107 (Range: 1–48)	1,269 (Range:1–45)
Mean time between first and last available VL measurement (months)	54.9	44.8	56.7	56.9	64.9
Number of patients with baseline VL	180	28	36	118	60
Undetectable VL at baseline					
**<50**	26	0	6	20	4
**<200**	33	0	8	25	4
**<500**	46	0	14	34	5
Number of patients with VL after LPV exposure	194	32	35	125	64
Undetectable VL at last available measurement					
**<50**	97	22	17	58	30
**<200**	118	25	24	69	34
**<500**	135	28	24	81	38
Undetectable VL at last available measurement^b^					
**<50**	1,739 (55.8%)	280 (63.9%)	409 (71.5%)	1,050 (49.8%)	556 (43.8%)
**<200**	1,822 (58.4%)	286 (65.3%)	421 (73.6%)	1,115 (52.9%)	564 (44.4%)
**<500**	2,054 (65.9%)	287 (65.5%)	440 (76.9%)	1,327 (63%)	673 (53%)
Undetectable VL measurement per patient^c^					
	Mean 15 (Range:0–48)	Mean 9 (Range:0–40)	Mean 12 (Range:0–37)	Mean 11 (Range: 0–29)	Mean 10.7 (Range: 0–38)
	59.6% IQR [22–92.3]	49.5% IQR [9.4–83.7]	68.3% IQR [50–91.7]	56.9% IQR [24.9–87.9]	44% IQR [0–81.1]

^**a**^No available data for 5 patients (1 in group 1, 1 in group 2 and 3 not ascribed to any group because of the lack of information).

^**b**^Undetectable VL considering values of <50, <200 and <500 HIV-1-RNA copies/ml, depending on available commercial VL technique used for HIV quantification at sample processing time.

^c^Undetectable VL as <500 HIV-1-RNA copies/ml; VL, viral load; IQR, interquartile range; no., number.

### Changes in lymphocyte counting after LPV/r exposure

We recovered baseline CD4 count from 159 (79.9%) patients and during LPV/r exposure in 192 (96.5%). When comparing baseline values *vs*. the last VL determination during LPV/r experience, a significant gain in the CD4 (+4.3% mean) and CD8 (+2.9% mean) rates in the three groups was observed. The highest gain was detected in group 1 patients (Table 2). CD4 percentage increase was only significant among patients from groups 1 and 3.

### DRM to PI and drug susceptibility to LPV/r

A total of 64 patients presented resistance information in PR (n = 64) or RT (n = 48) during LPV/exposure (Tables 4 and 5) and 35 of them also at baseline (S1 Table). Among the LPV/r exposed subjects, 27 (42.3%) carried DRM to PI, 28 (58.3%) to NRTI and 21 (43.7%) to NNRTI (Tables 4 and 5). Patients from group 3 presented the highest number of DRM to PI (52.2%), to NRTI (62.5%) and to NNRTI (46.9%).

**Table 4:** DRM presence at PR and predicted drug susceptibility in viruses from LPV/r-exposed patients with resistance data.

Patients	First LPV/r exposure	Patients with available data at PR^a^	Patients with major DRM at PR; mean DRM [IQR]	Predicted susceptibility to PI (%)							
				ATV/r	DRV/r	FPV/r	IDV/r	LPV/r	NFV	SQV/r	TPV/r
**Group 1** (n = 33)	In 1^st^ cART	8 (24.2%)	0 (0%)	100	100	100	100	100	62.5	100	87.5
**Group 2** (n = 37)	In 2^nd^ cART	10 (27%)	3 (30%); 2 [1–2.5]	80	90	80	80	90	40	90	90
**Group 3** (n = 126)	In ≥3^rd^ cART	46 (36.5%)	24 (52.2%); 2.7 [1–4]	56.5	78.3	50	52.2	58.7	45.6	60.9	67.4
**All** (n = 196)^b^	Any	64 (32.2%)	27 (42.3%); 2.6 [1–4]	65.6	82.8	60.9	62.5	68.7	46.8	70.3	73.4

^**a**^Available resistance data;

^b^Unknown LPV/r use in 3 patients; PI, protease inhibitor; PR, protease; DRM, Drug resistance mutation; r, ritonavir used for boosting; ATV/r, boosted-atazanavir; DRV/r, boosted-darunavir; FPV/r, boosted-fosamprenavir; IDV/r, boosted-indinavir; LPV/r, boosted-lopinavir; NFV, nelfinavir; SQV/r, boosted-saquinavir; TVR/r, boosted-tipranavir; RT, retrotrancriptase; NRTI, nucleoside reverse transcriptase inhibitors; NNRTI, non-NRTI; 3TC, lamivudine; ABC, abacavir; AZT, zidovudine; d4T, estavudine; DDI, didanosine; FTC, emtricitabine; TDF, tenofovir; EFV, efavirenz; ETR, etravirine; NVP, nevirapine; RPV, rilpivirine; cART, combination antiretroviral therapy; IQR, interquartile range.

**Table 5:** DRM presence at RT and predicted drug susceptibility in viruses from LPV/r-exposed patients with resistance data.

Patients	First LPV/r exposure	Patients with available data at RT^a^	Patients with DRM at RT; mean DRM [IQR]		Predicted susceptibility (%)										
					to NRTI							to NNRTI			
			to NRTI	to NNRTI	3TC	ABC	AZT	d4T	ddI	FTC	TDF	EFV	ETV	NVP	RPV
**Group 1** (n = 33)	In 1^st^ cART	8 (24.2%)	4 (50%);1.7 [1-3]	3 (37.5%);2 [1-3]	50	50	75	75	50	50	75	62.5	50	62.5	62.5
**Group 2** (n = 37)	In 2^nd^ cART	8 (24.2%)	4 (50%);2.5 [1.5-3.5]	3 (37.5%);1.3 [1-2]	62.5	50	75	75	50	62.5	62.5	75	75	75	75
**Group 3** (n = 126)	In ≥3^rd^ cART	32 (25.4%)	20 (62.5%);4.3 [3-6]	15 (46.9%);2.3 [1-3]	43.7	37.5	40.6	43.7	37.5	43.7	46.8	56.2	65.6	56.2	65.6
**All** (n = 196)^b^	Any	48 (24.5%)	28 (58.3%);3.7 [2-5.5]	21 (43.7%);2.1[1-2.5]	47.9	41.7	52.1	54.2	41.7	47.9	56.3	62.5	66.7	62.5	66.7

^a^Available resistance data;

^b^Unknown LPV/r use in 3 patients; PI, protease inhibitor; PR, protease; DRM, Drug resistance mutation; r, ritonavir used for boosting; ATV/r, boosted-atazanavir; DRV/r, boosted-darunavir; FPV/r, boosted-fosamprenavir; IDV/r, boosted-indinavir; LPV/r, boosted-lopinavir; NFV, nelfinavir; SQV/r, boosted-saquinavir; TVR/r, boosted-tipranavir; RT, retrotrancriptase; NRTI, nucleoside reverse transcriptase inhibitors; NNRTI, non-NRTI; 3TC, lamivudine; ABC, abacavir; AZT, zidovudine; d4T, estavudine; DDI, didanosine; FTC, emtricitabine; TDF, tenofovir; EFV, efavirenz; ETR, etravirine; NVP, nevirapine; RPV, rilpivirine; cART, combination antiretroviral therapy; IQR, interquartile range.

**Fig 1:**
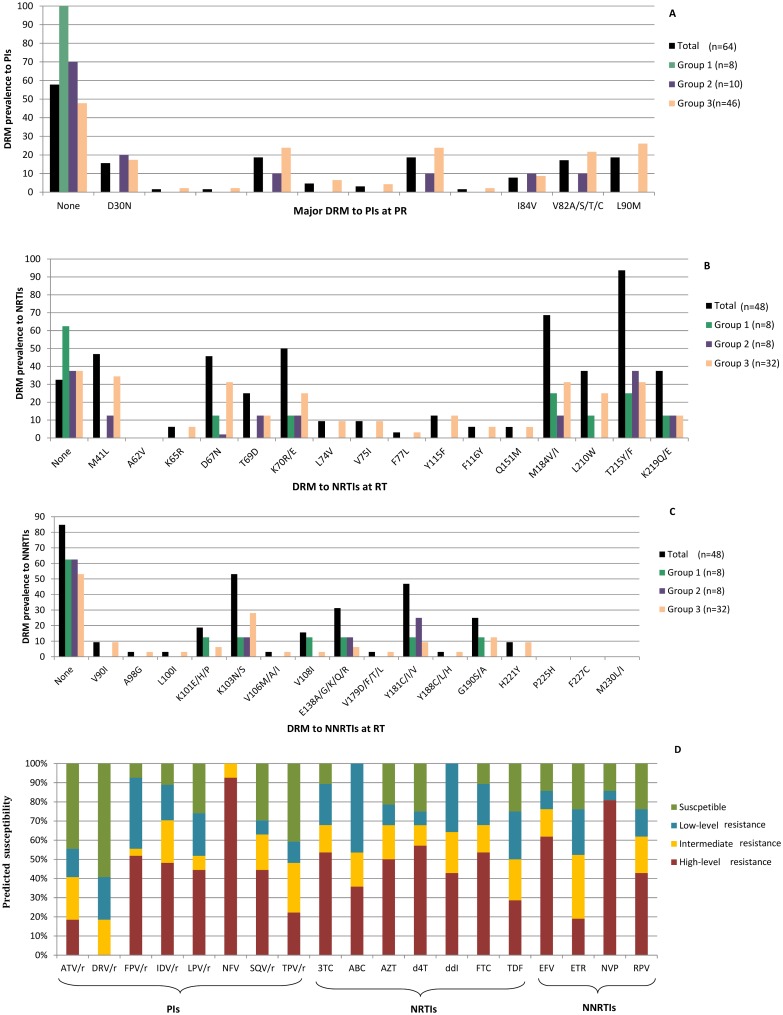
DRM in 64 LPV/r experienced paediatric patients and predicted drug-susceptibility in those carrying resistant viruses. Fig 1 legend: (A) DRM at PR associated with PI resistance in 64 HIV-1-infected paediatric patients with available PR resistant data (*PR* sequence or resistance profile to PI) during LPV/r exposure. (B) and (C) DRM at RT associated with NRTI or NNRTI resistance, respectively, in 46 HIV-1-infected paediatric patients with available *RT* sequence or resistance data to RT inhibitors during LPV/r experience. (D) Predicted susceptibility to antiretrovirals in viruses carrying DRM to IP (n = 27), to NRTI (n = 28) or to NNRTI (n = 21) according to Standford Algorithm. NRTI, nucleoside reverse transcriptase inhibitors; NNRTI, non-NRTI; r, ritonavir used for boosting; ATV/r, boosted-atazanavir; DRV/r, boosted-darunavir; FPV/r, boosted-fosamprenavir; IDV/r, boosted-indinavir; LPV/r, boosted-lopinavir; NFV, nelfinavir; SQV/r, boosted-saquinavir; TPV/r, boosted-tipranavir; 3TC, lamivudine; ABC, abcavir; AZT, zidovudine; d4T, estavudine; ddI, didanosine; FTC, emtricitabine; TDF, tenofovir; EFV, efavirenz; ETR, etravirine; NVP, nevirapine; RPV, rilpivirine.

Fig 1 summarizes the DRM in 64 LPV/r experienced paediatric patients in the cohort and in each group and the predicted drug-susceptibility in those 27 patients carrying DRM. Among those 64 patients with resistance data, seven (10.9%) were exposed to LPV/r as off-label therapy (1 in group 1 and 6 in group 3) and 4 (57.1%) of them carried DRM to PI.

The most common DRM to PI were D30N, M46IL, I54V, V82A and L90M in PR. D30N was the most frequently observed in virus from group 2 individuals and M46I/L, I54V, V82ASTC and L90M in group 3 patients (Fig 1A). Among those 27 viruses with DRM at PR, nelfinavir (NFV) was the most affected PI due to the presence of mutations D30N and L90M. However, darunavir-ritonavir (DRV/r), atazanavir-ritonavir (ATV/r) and tipranavir-ritonavir (TPV/r) presented the highest susceptibility (Fig 1D, Table 4), due to the absence or low rate of mutations I47V, I50V, I54ML, Q58E, T74P, L76V, V82LT, N83D and I84V in PR (Fig 1A). As expected, viruses presenting a lower number of DRM to PI at baseline and of previous ART regimens showed a higher predicted susceptibility to PI. However, despite the absence of viruses with DRM in group 1 patients, three patients showed low level resistance to NFV and one child to TPV/r (Table 4). We observed high resistance to fosamprenavir/ritonavir, indinavir/ritonavir, LPV/r, and saquinavir/ritonavir in nearly 50%, to TPV/r in 22.2% and to atazanavir/ritonavir (ATV/r) in 18.5% (Fig 1D). Among those 48 patients with available RT sequence or resistance profile, M184V and T215YF were the most common DRM to NRTI and K103N, Y181C and E138AGK to NNRTI (Fig 1B and 1C). Among those 28 patients with DRM to NRTI the most affected ARVs were abacavir, didanosine, lamivudine and emtrizabine (Fig 1D) and among those 21 carrying DRM to NNRTI, efavirenz and NVP, reported the lowest susceptibility (14.3%, both) (Fig 1D).

S1 Table shows the DRM to PI profile in the 35 children with available resistance data before and during LPV/r exposure, all with high basal viraemia and LPV/r exposure time.

### Risk factors for DRM development to PI

Supervised classification techniques are algorithms with high predictive power and designed to optimize the statistical classification procedures [29]. Supervised techniques using weka analysis identified the use of LPV/r as first ART and year of LPV/r start as the most important clinical factors under study predicting no DRM development to PI in our population (S2 Table). Accordingly, we observed the absence of DRM to PI in viruses from pediatric patients included in group 1.

## Discussion

The present study describes the clinical and virological features in patients from the Madrid Cohort of HIV-1-infected-children and adolescents exposed to LPV/r as different-line therapies during 2000–2014. It also reports the VL suppression and lymphocyte recovery after LPV/r exposure, the presence of DRM to three-drug classes and the predicted drug susceptibility among resistant viruses.

Current guidelines (United States and Europe) support the LPV-based regimen in combination with two NRTI in ARV-naïve children <3 years, and indicate that fosamprenavir, nelfinavir, stavudine, and unboosted atazanavir should not be used for initial therapy [2]. Despite LPV/r being the best choice for starting ART in children of less than three years in Spain, in the study cohort LPV/r was prescribed more frequently within rescue lines than in first-line treatments (83.2% *vs*. 16.8% cases) during 2000–2014 period. LPV/r was prescribed more frequently as part of the first cART regimen during 2008–2014 than during 2000–2007 (42.8% *vs*. 11%), probably due to several reasons: i) the lower number of newly infected infants in the first period requiring first therapy according to National guidelines; ii) The alternative efavirenz *vs*. PI use in children over 3 years with good expected adherence and the preferred ATV/r use due to its single dose use in children over 6 years, which had appeared in a higher number in the first period. iii) The withdrawal of Nelfinavir in 2007 by EMEA.

Although we observed an increase of CD4-T rate in the three groups, patients under first cART including LPV/r (group 1) with the highest number of CD4-T cells counts at baseline, showed significantly higher increases of CD4-T rate in the last available determination during LPV/r exp*os*ure, supporting previous studies showing that initiating cART at higher CD4-T cells counts maximizes the immunologic recovery [30]. We observed in the last available VL determination during LPV/r exposure, that 7 of each 10 patients with available data ach*ie*ved undetectable VL. Viremia reduction was higher (87.5%) in group 1 patients, supporting other studies showing that LPV/r-containing regimens result in potent and durable virological r*es*ponses in naïve children and in pretreated children [18]. Unfortunately, not all patients in the cohort achieved undetectable VL, since complete suppression of viral replication is more difficult to achieve in children than in adults due to several reasons, including a higher baseline VL, pharmacokinetic issues, the difficulty of permanent adherence because of the bad taste, complex regimens or inappropriate size of pills, and the frequent use of off-label ARV which can lead to under or overdosing [28], increasing the risk of DRM selection. As previously stated, 10.9% of children carrying DRM had been exposed to LPV/r as off-label ART.

Children failing first-line PI-based regimens do so with minimal development of PI resistance [31,32]. In fact, previous reports showed lower DRM appearance after first-line regimens including LPV/r based therapies *vs*. those including NFV [33] or NNRTI [34]. None of the studied children receiving LPV/r as first ART presented major DRM to PI, which explains the highest VL reduction after LPV/r exposure and preserved susceptibility to most PI observed in this group. As expected, group 3 patients showed the highest DRM rate to the three drug-families, explained by the longest exposure time to drugs. During 2008–2014, EMA approved alternative potent drugs for rescue ART regimens in children, such as new NRTI (tenofovir in 2012), new PI (TPVr and DRV/r in 2009 and ATV/r in 2010) and integrase inhibitor (raltegravir in 2013). According to our data, resistant viruses in LPV/r experienced children showed high susceptibility to TPV/r, ATV/r and DRV/r. Thus, those drugs would be effective after therapeutic failure events in these patients. Previous reports showed that the independent predictive factors related to virological success of LPV/r based ART regimens were plasma viraemia levels, previous PI use and the number of mutations reducing susceptibility to LPV/r at baseline [35,36].

In our study, 11 of the 37 children with no DRM to PI and resistance data before and after LPV/r exposure did not develop DRM at PR after a mean time of LPV/r exposure of 4.3 years (S1 Table). However, since none of them presented DRM to RT inhibitors, the high VL observed in the last available determination in three patients with known viremia would suggest bad adherence to treatment. The high rate of DRM to all drug classes among the 64 patients with available resistance data during LPV/r treatment is in agreement with the long history of therapy, frequent regimen switches and drug experience, and high off-labels ARV exposure, as previously reported [24, 28,37,38]. Some limitations to the study are the relatively late initiation of LPV/r in this cohort (9.5 years), the high rate of patients including LPV/r as at least third line treatment (with severe failure background), the high evolution of ARV options in children and adolescents during the study period and the late formal approval of LPV/r use in children. Moreover, only 64 of the 199 patients with LPV/r exposure during 2000–2014 presented available *pol* sequences or resistance profiles, favoured by the non recommendation of resistance testing in naïve subjects during clinical routine by the Spanish guidelines until 2007 [39] and to the low availability of samples before or after LPV/exposure. This study describes the clinical follow-up in a cohort of LPV/r experienced children and adolescents during 15 years in Spain. A higher VL reduction and better CD4 and CD8 recovery was observed when LPV/r was taken as part of a first-line regimen, reinforcing its use in children under 3 years old. LPV/r can also be a good alternative for rescue regimens in HIV-infected children with previous failure to NNRTI, and when other new PI are not available or present inadequate paediatric formulations for children less than 6 years. However, since most paediatric patients requiring a third ART line have been under PI exposure, LPV/r would not be a good candidate in this scenario.

## Supporting information

S1 Table DRM in 35 HIV-1 infected children with available data before and during LPV/r exposure. Patients without asterisk refers to the last baseline PR sequence before LPV/r exposure and marked with asterisk the last available PR sequence or resistance profile collected during LPV/r treatment until December 2011. Date: day/month/year. DRM, drug resistance mutations according to IAS-USA 2014 using Stanford′s HIVdb Algorithm (http://sierra2.stanford.edu/sierra/servlet/JSierra); PI, protease inhibitors; NA, data not available. Among these 35 patients, 11 carried wild type viruses in the analyzed baseline and post-LPV/r exposure sequences after a mean time of LPV/r exposure of 4.3 years. In 7 subjects infected by wild type viruses, major DRM to PI appeared after a mean time of LPV/r use of 7.3 years and a mean interval between sequences of 5.7 years. Seventeen patients were infected with viruses carrying major DRM to PI at baseline, maintaining resistant viruses at PR during their entire follow up in 10 cases or reverting to wild type viruses in 7 cases after a mean time of LPV/r use of 5.6 years. Five patients maintained the same DRM-PI profile in both sequences collected in a mean interval of 1.5 years and after a mean time of LPV/r exposure of 3.7 years, all with detectable and high VL at sampling time.
(DOCX)

S2 Table Risk factors for DRM development to protease inhibitors. CFS, Correlation Based Feature Selection; MI, Mutal information; Info, Information; no, number; LPV, lopinavir; ART, antiretroviral treatment. Predictive features selected by Univariate (Information gain and Gain Ration) and Multivariate (Correlation Feature Selection) analysis approach considering DRM as class variable. Selected attributes are displayed in ≥70% and threshold ≥ 0.1(10 folds).
(DOCX)
