# Supplementary material for: Impact of lopinavir-ritonavir exposure in HIV-1 infected children and adolescents in Madrid, Spain during 2000-2014
Source: PLoS One. 2017 Mar 28;12(3):e0173168. doi: 10.1371/journal.pone.0173168 (PMC5369685; doi:10.1371/journal.pone.0173168)
Supplement: S1 Table — Patients without asterisk refers to the last baseline PR sequence before LPV/r exposure and marked with asterisk the last available PR sequence or resistance profile collected during LPV/r treatment until December 2011. Date: day/month/year. DRM, drug resistance mutations according to IAS-USA 2014 using Stanford′s HIVdb Algorithm (http://sierra2.stanford.edu/sierra/servlet/JSierra); PI, protease inhibitors; NA, data not available. Among these 35 patients, 11 carried wild type viruses in the analyzed baseline and post-LPV/r exposure sequences after a mean time of LPV/r exposure of 4.3 years. In 7 subjects infected by wild type viruses, major DRM to PI appeared after a mean time of LPV/r use of 7.3 years and a mean interval between sequences of 5.7 years. Seventeen patients were infected with viruses carrying major DRM to PI at baseline, maintaining resistant viruses at PR during their entire follow up in 10 cases or reverting to wild type viruses in 7 cases after a mean time of LPV/r use of 5.6 years. Five patients maintained the same DRM-PI profile in both sequences collected in a mean interval of 1.5 years and after a mean time of LPV/r exposure of 3.7 years, all with detectable and high VL at sampling time. (DOCX) [file pone.0173168.s001.docx]

**Supplementary Table 1. DRM in 35 HIV-1 infected children with available data before and during LPV/r exposure.**

| **Patients** | **LPV/r group** | **Mean time**  **between sequences (months)** | **Mean time under LPV/r (months)** | **VL**  **(log)** | **DRM to PI (IAS-2014)** | |
| --- | --- | --- | --- | --- | --- | --- |
|  |  |  |  |  | **Major DRM** | **Number of minor DRM** |
| 1 | Group 1 | 4.9 | 16.6 | NA | NONE | 1 |
| 1* |  |  |  | NA | NONE | 1 |
| 2 | Group 1 | 70.5 | 102.9 | NA | NONE | 1 |
| 2* |  |  |  | NA | NONE | 2 |
| 3 | Group 2 | 20.1 | 18 | NA | NONE | 0 |
| 3* |  |  |  | NA | NONE | 0 |
| 4 | Group 3 | 9.1 | 9.3 | NA | NONE | 3 |
| 4* |  |  |  | NA | NONE | 4 |
| 5 | Group 3 | 13.2 | 43.1 | NA | NONE | 2 |
| 5* |  |  |  | NA | NONE | 1 |
| 6 | Group 3 | 23.8 | 31.6 | NA | NONE | 1 |
| 6* |  |  |  | NA | NONE | 3 |
| 7 | Group 3 | 40.3 | 89.1 | NA | NONE | 2 |
| 7* |  |  |  | NA | NONE | 2 |
| 8 | Group 3 | 46.9 | 42.5 | 4.8 | NONE | 3 |
| 8* |  |  |  | 3.8 | NONE | NA |
| 9 | Group 3 | 54.9 | 48.5 | 4.5 | NONE | 2 |
| 9* |  |  |  | 4.7 | NONE | 5 |
| 10 | Group 3 | 74.7 | 54.6 | NA | NONE | 0 |
| 10* |  |  |  | NA | NONE | 1 |
| 11 | Group 3 | 113.1 | 108.4 | 2.5 | NONE | 1 |
| 11* |  |  |  | 5.4 | NONE | 0 |
| 12 | Group 3 | 5.4 | 57.9 | 4.5 | D30N | 2 |
| 12* |  |  |  | 2.3 | D30N | 2 |
| 13 | Group 3 | 6.9 | 10.2 | 5 | D30N | 3 |
| 13* |  |  |  | 6 | D30N | 5 |
| 14 | Group 3 | 12.7 | 49.2 | NA | D30N, L90M | 5 |
| 14* |  |  |  | NA | D30N, L90M | 5 |
| 15 | Group 3 | 13.5 | 39.5 | 4.1 | L90M | 6 |
| 15* |  |  |  | 4.4 | L90M | 6 |
| 16 | Group 3 | 55.2 | 64.9 | NA | M46I, I84V, L90M | 2 |
| 16* |  |  |  | NA | M46I, I84V, L90M | 2 |
| 17 | Group 3 | 23.2 | 26.5 | 5.1 | M46I, V77I, I84V, L90M | 4 |
| 17* |  |  |  | 5.2 | M46I, I47V, I54V, I84V, L90M | 10 |
| 18 | Group 3 | 25.1 | 48.1 | NA | M46I, L90M | 6 |
| 18* |  |  |  | NA | M46I, I54V, V82A, L90M | 9 |
| 19 | Group 3 | 39.9 | 87.5 | 4.7 | V32I, M46I, V82A, I84V, L90M | 6 |
| 19* |  |  |  | 4.8 | V32I, M46I, I54V, V82A, I84V, L90M | 5 |
| 20 | Group 3 | 53.2 | 57 | 4.9 | N88S | 4 |
| 20* |  |  |  | 4.4 | M46I, I54V, V82A | 4 |
| 21 | Group 2 | 63.3 | 97.9 | 4.5 | NONE | 0 |
| 21* |  |  |  | 1.6 | D30N | 3 |
| 22 | Group 3 | 9.6 | 53.1 | 5.2 | NONE | 0 |
| 22* |  |  |  | 2.6 | D30N | 2 |
| 23 | Group 3 | 45.4 | 84.9 | 4.7 | NONE | 5 |
| 23* |  |  |  | 4.1 | M46I, V82A,I54V | 8 |
| 24 | Group 3 | 57.7 | 57.3 | 4.9 | NONE | 2 |
| 24* |  |  |  | 5.2 | I54V, V82A, L90M | 8 |
| 25 | Group 3 | 80.9 | 90.8 | 4.6 | NONE | 2 |
| 25* |  |  |  | 3.4 | D30N, M46I | NA |
| 26 | Group 3 | 90.7 | 94.3 | NA | NONE | 2 |
| 26* |  |  |  | NA | D30N, N88D | 2 |
| 27 | Group 3 | 130.1 | 134.2 | NA | NONE | 0 |
| 27* |  |  |  | NA | M46IM, L90LM | 5 |
| 28 | Group 3 | 53.9 | 8.3 | NA | D30N, N88D | 3 |
| 28* |  |  |  | NA | D30N | 7 |
| 29 | Group 1 | 11.9 | 54.2 | NA | V82AV | 2 |
| 29* |  |  |  | NA | NONE | 4 |
| 30 | Group 2 | 5.7 | 129.7 | NA | D30N, M46I | 3 |
| 30* |  |  |  | NA | NONE | 3 |
| 31 | Group 3 | 8.4 | 0.63 | 4.3 | D30N | 4 |
| 31* |  |  |  | 4.8 | NONE | 2 |
| 32 | Group 3 | 16.8 | 14.5 | NA | D30N | 2 |
| 32* |  |  |  | NA | NONE | 0 |
| 33 | Group 3 | 30.9 | 128.2 | 3.8 | M46I | 3 |
| 33* |  |  |  | 2.1 | NONE | 1 |
| 34 | Group 3 | 46.7 | 65.9 | NA | D30N, M46I | 4 |
| 34* |  |  |  | NA | NONE | 1 |
| 35 | Group 3 | 50.4 | 74.9 | NA | M46L, V82A, L90M | 9 |
| 35* |  |  |  | NA | NONE | NA |

**LEGEND Supplementary Table 1:**

Patients without asterisk refers to the last baseline PR sequence before LPV/r exposure and marked with asterisk the last available PR sequence or resistance profile collected during LPV/r treatment until December 2011. DRM, drug resistance mutations according to IAS-USA 2014 using Stanford´s HIVdb Algorithm (http://sierra2.stanford.edu/sierra/servlet/JSierra); PI, protease inhibitors; NA, data not available.

Among these 35 patients, 11 carried wild type viruses in the analyzed baseline and post-LPV/r exposure sequences after a mean time of LPV/r exposure of 4.3 years. In 7 subjects infected by wild type viruses, major DRM to PI appeared after a mean time of LPV/r use of 7.3 years and a mean interval between sequences of 5.7 years. Seventeen patients were infected with viruses carrying major DRM to PI at baseline, maintaining resistant viruses at PR during their entire follow up in 10 cases or reverting to wild type viruses in 7 cases after a mean time of LPV/r use of 5.6 years. Five patients maintained the same DRM-PI profile in both sequences collected in a mean interval of 1.5 years and after a mean time of LPV/r exposure of 3.7 years, all with detectable and high VL at sampling time.
