# Supplementary material for: Impact of lopinavir-ritonavir exposure in HIV-1 infected children and adolescents in Madrid, Spain during 2000-2014
Source: PLoS One. 2017 Mar 28;12(3):e0173168. doi: 10.1371/journal.pone.0173168 (PMC5369685; doi:10.1371/journal.pone.0173168)
Supplement: S2 Table — CFS, Correlation Based Feature Selection; MI, Mutal information; Info, Information; no, number; LPV, lopinavir; ART, antiretroviral treatment. Predictive features selected by Univariate (Information gain and Gain Ration) and Multivariate (Correlation Feature Selection) analysis approach considering DRM as class variable. Selected attributes are displayed in ≥70% and threshold ≥ 0.1(10 folds). (DOCX) [file pone.0173168.s002.docx]

**Supplementary Table 2. Risk factors for DRM development to protease inhibitors.**

|  | **Univariate** | | **Multivariate (CFS)** |
| --- | --- | --- | --- |
| **Selected Variable** | **MI Info. Gain** | **MI Info. Gain Ratio** | **no. of folds %** |
| **HIV diagnosis year** | 0.196 | 0.292 | 100% |
| **Year of LPV/r start** | 0.181 | 0.252 | 100% |
| **Year of any ART start** | 0.164 | 0.165 | 90% |
| **CD4 T-cells** | 0.156 | - | 100% |
| **Sequence year** | 0.138 | 0.192 | 90% |
| **LPV/r as first ART** | 0.123 | 0.287 | 80% |

**Legend Supplementary Table 2:**

CFS, Correlation Based Feature Selection; MI, Mutal information; Info, Information; no, number; LPV, lopinavir; ART, antiretroviral treatment. Predictive features selected by Univariate (Information gain and Gain Ration) and Multivariate (Correlation Feature Selection) analysis approach considering DRM as class variable. Selected attributes are displayed in ≥70% and threshold ≥ 0.1(10 folds)**.**
